# The 1.78-kb insertion in the 3′-untranslated region of *RXFP2* does not segregate with horn status in sheep breeds with variable horn status

**DOI:** 10.1186/s12711-016-0256-3

**Published:** 2016-10-19

**Authors:** Gesine Lühken, Stefan Krebs, Sophie Rothammer, Julia Küpper, Boro Mioč, Ingolf Russ, Ivica Medugorac

**Affiliations:** 1 Department of Animal Breeding and Genetics, Justus Liebig University of Gießen, Ludwigstrasse 21a, 35390 Giessen, Germany; 2 Laboratory for Functional Genome Analysis (LAFUGA), Gene Center Munich, LMU Munich, Feodor-Lynen-Strasse 25, 81377 Munich, Germany; 3 Chair of Animal Genetics and Husbandry, LMU Munich, Veterinaerstrasse 13, 80539 Munich, Germany; 4 Department of Animal Science and Technology, Faculty of Agriculture, University of Zagreb, Svetošimunska cesta 25, 10000 Zagreb, Croatia; 5 Tierzuchtforschung e.V. München, Senator-Gerauer-Strasse 23, 85586 Poing, Germany

## Abstract

**Background:**

The mode of inheritance of horn status in sheep is far more complex than a superficial analysis might suggest. Observations, which were mostly based on crossbreeding experiments, indicated that the allele that results in horns is dominant in males and recessive in females, and some authors even speculated about the involvement of more than two alleles. However, all recent genome-wide association analyses point towards a very strong effect of a single autosomal locus on ovine chromosome 10, which was narrowed down to a putatively causal insertion polymorphism in the 3′-untranslated region of the *relaxin/insulin-like family peptide receptor 2* gene (*RXFP2*). The main objective of this study was to test this insertion polymorphism as the causal mutation in diverse sheep breeds, including breeds with a variable and/or sex-dependent horn status.

**Results:**

After re-sequencing a region of about 246 kb that covered the *RFXP2* gene and its flanking regions for 24 sheep from six completely horned and six completely polled breeds, we identified the same insertion polymorphism that was previously published as segregating with horn status in these breeds. Multiplex PCR genotyping of 489 sheep from 34 breeds and some crosses between sheep breeds showed a nearly perfect segregation of the insertion polymorphism with horn status in sheep breeds of Central and Western European origin. In these breeds and their crossings, heterozygous males were horned and heterozygous females were polled. However, this segregation pattern was not, or at least not completely, reproducible in breeds with sex-dependent and/or variable horn status, especially in sheep that originated from even more southern European regions and from Africa. In such breeds, we observed almost all possible combinations of genotype, sex and horn status phenotype.

**Conclusions:**

The 1.78-kb insertion polymorphism in the 3′-untranslated region of *RXFP2* and SNPs in the 3′-UTR, exon 14 and intron 11 of this gene that we analyzed in this study cannot be considered as the only cause of polledness in sheep and are not useful as a universal marker to define the genetic horn status in sheep.

**Electronic supplementary material:**

The online version of this article (doi:10.1186/s12711-016-0256-3) contains supplementary material, which is available to authorized users.

## Background

In sheep, horn status is influenced by sex and varies between breeds. Castle [1] categorized sheep breeds into three types, i.e. (1) both sexes carry horns but those of the females are much smaller (similar to the horn status of wild sheep in Central Asia); (2) males have well-developed horns, females are polled (similar to the horn status of most mouflons that originate from Sardinia [2]); and (3) both sexes are polled (this is the case for the majority of domestic sheep breeds). However, regarding the horn status, many sheep breeds do not fall into these three categories. For example, in several breeds such as Soay, Bündner Oberland and Sakiz, and also in the mouflons that originate from Corsica [2, 3], males are strictly horned and females may or may not be horned, while the reverse is observed in other breeds, e.g. Altamurana and Red Karaman, with females being strictly polled and males having or not having horns. Finally, there are some breeds in which the occurrence of horns varies both in males and females, for example in some strains of Steinschaf and of Pramenka such as Travnička Pramenka [4].

Another important and complicating feature of the horn phenotype in some breeds of sheep is the development of knobs and scurs, which is sex-dependent and breed-dependent. Warwick and Dunkle [5] describe knobs as protrusions from the scull that resemble horn cores, except that they are usually less than 2.5 cm (1 in.) high and covered with skin. Scurs have a horn-like covering but are smaller than normal horns and irregular in shape. According to these authors, knobs and scurs are observed in females of Merino-type breeds (including the Rambouillet breed) in which males are horned and females are polled. In contrast, in breeds in which both sexes are polled (e.g. Shropshire, Southdown, and Suffolk), depressions in the skull instead of horn cores are observed in both males and females. It should be mentioned at this point that, in some publications, animals with knobs or scurs are referred to as “horned”, whereas in others, they are referred to as “polled”.

The mode of inheritance of the horn phenotype in sheep is far more complex than a superficial analysis might suggest. Already more than 100 years ago, several studies showed that in crosses between Dorset Horn (a breed in which both sexes are strictly horned) and completely hornless (polled) breeds [6–8], only the male offspring inherited the horned phenotype. Based on these observations, Wood [7] stated that horns are dominant in male sheep and recessive in female sheep. Results from subsequent studies in the same and other breeds also suggested that the effects of the horned and polled alleles differed between male and female sheep, e.g. [9–13]. More recently, Johnston et al. [14] concluded that in the Soay breed, the mode of inheritance of horns was additive in ewes and dominant in rams.

In 1912, Arkell and Davenport [15] suggested the existence of a sex chromosome-linked inhibitor of horn development, but this hypothesis did not gain common acceptance. In a back-cross between one F_1_ Dorset × Rambouillet ram and Merino and Rambouillet ewes that carried knobs, Warwick and Dunkle [5] observed eight individuals with horns and 14 with knobs among the female progeny, but no individual showed depressions in the skull instead of horn cores. Because of this absence of polled sheep with depressions, they concluded that the three genes responsible for the absence of horns or polledness i.e. *H*, for Dorset horns i.e.  *H'* (responsible for horns in strictly horned breeds), and for Merino (and Rambouillet) horns i.e. *h* (responsible for sex-dependent horns), are not independent genes but three alleles at one locus. In the Merino breed, polledness was observed to be produced by an incompletely dominant gene, *P*. Polled Merinos were supposed to be *PP* or *Pp*, while Merino rams with horns and ewes with knobs or short scurs were supposed to be *pp* [15]. Dolling [16] suggested that a third gene, *P′*, either an allele of *P*/*p* or closely linked to *P* and *p*, was also involved in the horn phenotype since Peppin Merino ewes carry horns although in Merino breeds ewes are polled. Based on the results from a series of crosses between Boder Leicester and Australian Merino sheep, Dolling [9] concluded that the genes that cause polledness in these two breeds were either closely-linked alleles of two loci or were identical and favored the last interpretation.

In 1996, Montgomery et al. [17] showed that the horn status in sheep is controlled by a locus on the proximal end of sheep chromosome OAR10 (OAR for *Ovis aries* chromosome), and since then several mapping studies have confirmed and gradually narrowed down the location of the responsible region [18–20]. As a preliminary result, Pickering et al. [20] reported that an approximately 3-kb retrotransposed insertion in the 3′ untranslated region (UTR) of an unnamed candidate gene was present only in polled animals. Several whole-genome association analyses using the Illumina ovine 50K SNP (single nucleotide polymorphism) chip to genotype different sheep breeds identified several SNPs that were strongly associated with horn status and located between positions 29.36 and 29.51 Mb on OAR10 (the positions refer to the sheep genome assembly Oar_v3.1 and correspond with a region on chromosome 10 (NC_019467.2) between 29.34 and 29.49 Mb of the sheep genome assembly Oar_v4.0); some of these SNPs were immediately adjacent to or even located within the *relaxin/insulin-like family peptide receptor 2* gene (*RXFP2*) [14, 21, 22]. SNP OAR10_29511510.1 was the third most strongly associated SNP in the study of Johnston et al. [14] and is located in intron 11 of *RXFP2*.

Sequence analyses of the promoter region and of all the exons and their boundaries of the *RXFP2* gene on DNA samples from male and female Tan sheep (that include horned males and horned, scurred or polled females) and from males and females of the strictly polled Suffolk breed allowed the detection of several additional novel SNPs [23]. Four of these SNPs were located in the coding sequence of *RXFP2* and two of these caused amino acid substitutions. None of the SNPs segregated with horn status through a Mendelian mode of inheritance in either of these breeds. However, one synonymous SNP in exon 14 (c.1125A>G) was found to be a potential indicator of the presence or absence of horns in Tan sheep [23].

Initially, the objective of our study was to test the association between horn status in sheep and the 3-kb insertion polymorphism that was previously identified by Pickering et al. [20] but not described in more detail and to prove the need for developing a mapping design across a wide range of sheep breeds. In order to retrieve the ‘Pickering’ insertion and to test for its postulated mode of inheritance and the possible existence of additional polymorphisms in the target region, we re-sequenced a 246-kb region that included the *RFXP2* gene and its flanking regions in sheep from strictly horned and strictly polled breeds. During the course of our work, Wiedemar and Drögemüller [24] in a study on several Swiss sheep breeds rediscovered most possibly the same insertion and showed that it was associated with horn status. Once our sequencing results had confirmed the results of Pickering et al. [20] and of Wiedemar and Drögemüller [24], our primary aim was to test the segregation of this insertion polymorphism in several sheep breeds and in crosses between breeds with different horn statuses, in particular breeds with a variable and/or sex-dependent horn status. Additional SNPs within and near the *RXFP2* gene were tested on a reduced animal dataset to demonstrate the absence of any direct causal relationship of this gene with polledness in a wide range of sheep breeds.

## Methods

### Animal samples and DNA extraction

Collection of the blood samples used in this study was performed exclusively by local veterinarians during regular quality control of breeding records (e.g. paternity testing) on the farms, thus no authorization from the ethics committee was required.

**Fig. 1:**
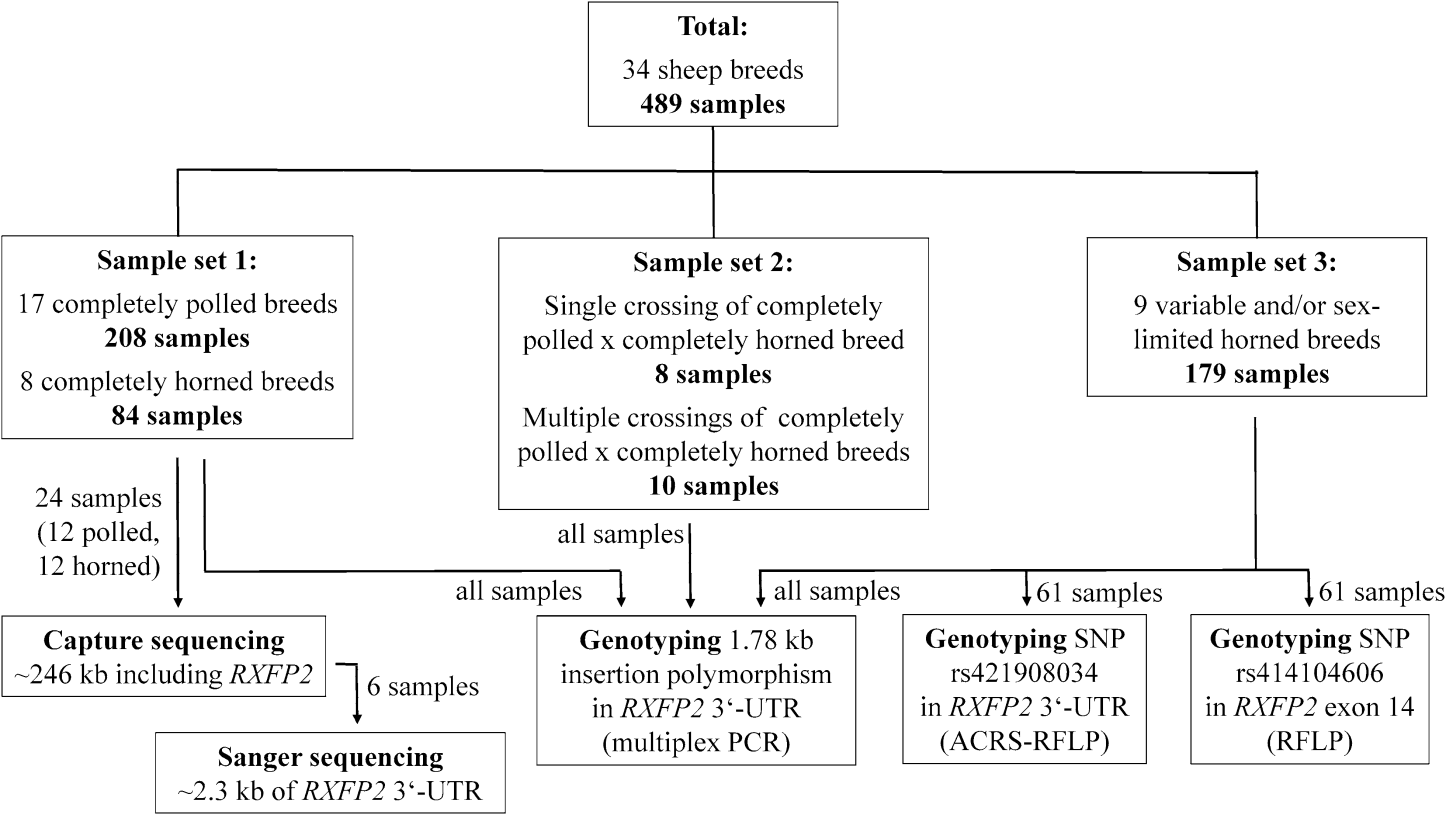
Overview of samples and molecular genetic methods used

Extraction of genomic DNA from peripheral blood was done by using either a modified high salt method [25] or a commercial spin column kit (QIAamp DNA blood mini, Qiagen, Hilden). DNA was obtained from 489 pure and crossbred sheep, which were sampled in Germany except for three breeds or strains that originated from Croatia (Cres sheep, Krk sheep and Travnička Pramenka). However, many of the sheep bred and sampled in Germany have their genetic origin in various regions of Europe. Figure 1 shows an overview of the sample sets used and the number of samples per set analyzed with different molecular genetic methods.

The first sample set included 208 samples from 17 completely polled and 84 samples from eight completely horned breeds (see details in Table 1). Sample set 2 included samples from 18 sheep that originated either from one cross between a completely polled breed and a completely horned breed or from multiple crosses between completely polled and completely horned breeds (details in Table 2). The third sample set consisted of 179 samples from nine breeds that have heterogeneous horn statuses, i.e. that ranged from breeds in which all males are horned and all females are (usually) polled to breeds in which both males and females can be polled or horned (details in Table 3). Some breeds also included individuals with scurs or horn rudiments. All sheep of sample sets 2 and 3 were phenotyped for horn status at an age at which horns are usually developed. In general, the names of the sheep breeds mentioned in this article refer to Mason and Porter [3], except for the breeds Travnička Pramenka [4], Alpines Steinschaf and Cameroon sheep (dad.fao.org).

**Table 1:** Genotypes at the insertion polymorphism in the 3′-UTR of *RXFP2* for sheep from completely polled and completely horned breeds (sample set 1)

Horn status of breeds	Breed	Genotypes (n)		
		*anc*/*anc*	*anc*/*der*	*der*/*der*
Completely polled	Barbados Black Belly		3^a^	7
	Bentheimer^b^			4
	Charollais			3
	Coburger			18
	East Friesian (White)^b^			20
	German Blackheaded Mutton			20
	German Brown Mountain			4
	German White Mountain^b^			20
	Ile de France			3
	Merinolandschaf			20
	Pomeranian Coarsewool^b^		1^a^	18
	Rhön^b^			20
	Rouge du Roussillon			4
	Shropshire			4
	Suffolk			17
	Texel			20
	White Polled Heidschnucke^b^			2
Completely horned	Grey Horned Heidschnucke^b^	26		
	Racka	7		
	Roux du Valais^b^	2		
	Scottish Blackface^b^	18		
	Soay^c^	9		
	Valais Blacknose^b^	18		
	White Horned Heidschnucke^b^	2		
	Wiltshire Horn^b^	2		

*anc* ancestral allele, *der* derived allele (with the 1.78-kb insertion), *n* number

^a^Female(s)

^b^Two animals of this breed were also used for sequencing

^c^Originating from a completely horned population

**Table 2:** Genotypes at the insertion polymorphism in the 3′-UTR of *RXFP2 *for crossbreds between completely polled and completely horned sheep breeds (sample set 2)

Cross	Crossed breeds	Horn status of the animals	Sex	Genotypes (n)		
				*anc*/*anc*	*anc*/*der*	*der*/*der*
Single cross	East Friesian (white) × Grey Horned Heidschnucke	Horned	Male		3	
		Polled	Female		3	
	Merinolandschaf × Grey Horned Heidschnucke	Horned	Male		2	
Multi cross	Completely polled breeds × completely horned breeds	Horned	Female	3		
		Polled	Female		4	2
		Horned^a^	Female		1	

*anc* ancestral allele, *der* = derived allele (with the 1.78-kb insertion), *n* number

^a^Horn rudiments

**Table 3:** Genotypes at the insertion polymorphism in the 3′-UTR of *RXFP2* for sheep from breeds with variable horn status (sample set 3)

Horn status of breed	Breed	Sheep (n)	Horn status of animals	Sex	Genotypes (n)		
					*anc*/*anc*	*anc*/*der*	*der*/*der*
Variable horn status of males and females	Bavarian forest	9	Horned	Male	6	3	
		3	Horned	Female	1	2	
		1	Horned^a^	Female		1	
		1	Polled	Male			1
		4	Polled	Female		3	1
		2	Scurred	Male		2	
		3	Scurred	Female		3	
Variable horn status of males and females	Alpines Steinschaf	6	Horned	Male	6		
		6	Horned	Female	6		
		2	Horned^b^	Male		2	
		1	Polled	Male		1	
		8	Polled	Female		7	1
		1	Scurred	Male			1
Males horned, females variable	Walachenschaf	5	Horned	Male	4	1	
		5	Horned	Female	5		
		3	Horned^b^	Female	2	1	
Males horned, females usually polled	Cres sheep	2	Polled	Female	1	1	
Males horned, females usually polled	Krk sheep	3	Polled	Female			3
Variable horn status of males and females	Travnička Pramenka	2	Horned	Male	2		
		12	Horned	Female	12		
		7	Horned^b^	Female	6	1	
		5	Polled	Male		4	1
		15	Polled	Female	13	2	
		2	Scurred	Female	2		
Variable horn status of males and females	Bovec-like (Krainer Steinschaf)	5	Horned	Male			5
		5	Horned	Female			5
		1	Horned^b^	Male			1
		3	Polled	Male			3
		8	Polled	Female			8
		1	Scurred	Female			1
Variable horn status of males and females	Dorper	8	Horned	Male			8
		6	Polled	Male			6
		3	Scurred	Male			3
		7	Scurred	Female			7
Males horned, females polled	Cameroon sheep	9	Horned	Male	11		
		1	Polled	Male		1	
		13	Polled	Female	10	2	

*anc* ancestral allele, *der* = derived allele (with 1780 bp-insertion), n number

^a^Right horned, left scurred

^b^Horn rudiments

### Sequence capture and sequencing of the *RXFP2* gene and its flanking regions

A region of about 246 kb (NC_019467.2:29,331,000-29,577,000, Oar_v4.0) on OAR10 that included the complete *RXFP2* gene (about 60 kb), the region flanking exon 1 and its 3′-UTR (about 185 kb) was sequenced. For this purpose, 24 DNA samples that included two individuals from six completely polled breeds (Bentheimer, East Friesian, German White Mountain, Pomeranian Coarsewool, Rhön, and White Polled Heidschnucke) and six completely horned breeds (Grey Horned Heidschnucke, Roux du Valais, Scottish Blackface, Valais Blacknose, White Horned Heidschnucke, and Wiltshire Horn) were selected from sample set 1 (Fig. 1).

For the generation of sequencing libraries, 1 µg of genomic DNA was sonicated (Bioruptor, Diagenode, Liege) for 25 cycles (30 s on/off at “low” intensity) and processed with the Accel-DNA 1S kit (Swift Biosciences, Ann Arbor) according to the manufacturer’s instructions. The resulting barcoded whole-genome libraries were pooled in equimolar amounts and hybridized to a genomic tiling array (Agilent 244 k capture Array, Agilent, Santa Clara; custom designed by e-array, repeat-masked, 2-bp tiling). Briefly, the libraries were hybridized for 65 h at 65 °C, washed and eluted with nuclease-free water for 10 min at 95 °C. The eluted DNA was concentrated in a vacuum centrifuge, amplified by PCR (10 cycles at 98 °C for 15 s, 65 °C for 30 s, and 72 °C for 30 s) and purified on Ampure XP beads. The target-enriched libraries were sequenced on a Hiseq 1500 instrument (Illumina, SanDiego) in paired-end mode with a read length of 100 nt for each read. Reads were de-multiplexed and mapped to the reference sheep genome (Oar_v4.0) using the BWA software package [26]. After removal of PCR duplicates, variants were called by using the software VarScan, which is optimized to detect SNPs and short indels, and then searched for potential candidate variants that co-segregated with the horned phenotype but were absent in polled animals. The insertion polymorphism was identified manually using the Integrative Genomics Viewer software (IGV, http://www.software.broadinstitute.org/software/igv/). Because of the manual process used for the IGV analyses, they were very time-consuming, thus we focused mainly on the sequence data of some previously published regions within the 246-kb region.

To identify the chromosomal segment that segregates with polledness in sheep, all informative SNPs [with a minor allele frequency (MAF) higher than 0.05] were filtered out from the data obtained in the VarScan process. For each SNP, the major allele that was present in the 12 polled animals, i.e. the case group, was identified and their average frequencies were estimated separately for the polled and horned groups using 20-kb sliding windows. Finally, the *F*_ST_-values were estimated based on the allele frequencies of the same 20-kb windows and graphically represented. The identical by descent (IBD) region was then defined as the region showing *F*_ST_-values higher than 0.3159 (mean *F*_ST_-value plus one standard deviation).

### Determination of the exact sequences of the identified variations in the 3′-UTR of *RXFP2* by Sanger sequencing

In order to determine the exact sequence of two variants that had been identified in the 3′-UTR of *RXFP2* by targeted sequencing, primers F1 and R1 (see Additional file 1: Table S1) and standard PCR conditions were used to amplify two fragments of about 500 bp from the DNA of two individuals from completely horned breeds (Roux du Valais and Valais Blacknose) and of about 2.3 kb from the DNA of two individuals from completely polled breeds (Bentheimer and Rhön). These PCR products were sequenced (Eurofins Genomics, Ebersberg) on both strands using the same PCR primers. In order to obtain the complete sequence of the insertion, additional sequencing reactions with primers F3 and F4 (see Additional file 1: Table S1) were performed on PCR products from polled sheep only. The resulting chromatograms were analyzed with the software *ChromasPro* version 1.33 (Technelysium Pty Ltd, Tewantin) and compared with the GenBank reference sequence of ovine *RXFP2*, NC_019467.2 (based on the sheep genome assembly Oar_v4.0).

To distinguish between ancestral and derived alleles, we performed multiple sequence alignment of the region that surrounds the variable site in the 3′-UTR of *RXFP2* of whole-genome sequences of seven *Bovidae* using NCBI’s blast server (http://www.blast.ncbi.nlm.nih.gov/Blast.cgi) with default settings (Megablast).

### Genotyping of ancestral and derived alleles in the 3′-UTR of *RXFP2* by multiplex PCR

To determine the ancestral and derived alleles of the 3′-UTR of *RXFP2* that segregated with horn status in the 24 sequenced samples of sample set 1, a multiplex PCR was designed and applied to all 489 samples of sample sets 1, 2 and 3: if the insertion was present, primer pairs F1/R1, F1/R2 and F2/R1 (see Additional file 1: Table S1) were expected to produce fragments of 2286, 389 and 676 bp, respectively, but if the individual carried the ancestral allele (without the insertion), primer pair F1/R1 was expected to amplify a fragment of only 506 bp. PCR amplifications were carried out according to the manufacturer’s standard protocol for the Promega Go Taq polymerase (Promega, Mannheim) in a 15-µL reaction volume that contained the four primers (6 pmol of primer F2, 4 pmol of each of the other three primers), 1.0 mM MgCl_2_ and 10 to 50 ng of genomic DNA. PCR conditions were as follows: 2 min at 95 °C followed by 33 cycles at 95 °C for 30 s, 60 °C for 45 s and 72 °C for 1 min, and a final elongation step at 72 °C for 10 min. After electrophoresis in a 1.5 % agarose gel stained with Midori Green Advance as recommended by the manufacturer (Biozym Scientific, Hessisch Oldendorf), PCR products were detected using UV light.

### Genotyping of a SNP in the 3′-UTR of *RXFP2* by amplification-created restriction site-restriction fragment length polymorphism (ACRS-RFLP) analysis

A 104-bp fragment that included an A/G substitution in the 3′-UTR of *RXFP2* at position NC_019467.2:29,432,846 (rs421908034) was amplified using the forward primer 5′-CAAGCCAAAAAGGTGAATGG-3′ and the reverse primer 5′-GTGGAGCAGCAGCTTTGAAAT-3′. The reverse primer included a mismatch nucleotide (underlined) to create a restriction site for the restriction enzyme *Hpy188*I in the presence of allele *G*. PCR amplifications were carried out according to the manufacturer’s standard protocol for the Promega Go Taq polymerase (Promega, Mannheim) in a 25-µL reaction volume that included forward and reverse primers (10 pmol of each), 2.0 mM MgCl_2_ and 10–50 ng of genomic DNA. PCR conditions were as follows: 1.5 min at 95 °C followed by 35 cycles at 95 °C for 15 s, 56 °C for 20 s and 72 °C for 30 s, and a final elongation step at 72 °C for 5 min. After electrophoresis on a 3.0 % agarose gel stained with Midori Green Advance (as above), PCR products were detected using UV light. PCR products of 61 samples from sample set 3 (that comprised the Wallachenschaf, Dorper, and Cameroon breeds) were incubated with *Hpy188*I as recommended by the manufacturer (New England Biolabs, Ipswich, MA). Expected sizes of the fragments after digestion were 104 bp if allele *A* was present and 82 and 22 bp if allele *G* was present.

### Genotyping of a SNP in exon 14 of *RXFP2* by restriction fragment length polymorphism (RFLP) analysis

A fragment that included the synonymous SNP c.1125A>G in exon 14 of *RXFP2* was amplified as described by Wang et al. [23]. However, based on the ovine genome sequence, the resulting fragment was expected to include 754 bp instead of the 765 bp that were indicated by Wang et al. [23]. PCR products of 61 samples from sample set 3 (that included the Walachenschaf, Dorper and Cameroon breeds) were incubated with *Hpy188*I (as above). Expected sizes of the fragments after digestion were 726 and 28 bp if allele *A* was present and 429, 297 and 28 bp if allele *G* was present.

### Analysis of genotyping data for SNP OAR10_29511510.1 in breeds with a fixed horn status

The International Sheep Genomics Consortium (ISGC) [22] produced genotyping data for 49,034 SNPs, including SNP OAR10_29511510.1 (rs413264476) that is located within intron 11 of *RXFP2*, on 2819 individuals that represent a diverse collection of 74 sheep breeds. However, the horn status of each individual is not available for this data. Thus, among the genotyped breeds, we selected those with a fixed, breed-specific horn status, which included breeds that are completely polled or completely horned as well as breeds with a fixed sex-dependent horn status (all males horned, all females polled). The horn status of the breeds was retrieved from relevant publications such as Mason and Porter [3], online breed databases (e.g. DAD-IS, dad.fao.org) and information provided by breeding organizations. Breed names were those indicated by the ISGC.

## Results

### Sequence capture and Sanger sequencing results

**Fig. 2:**
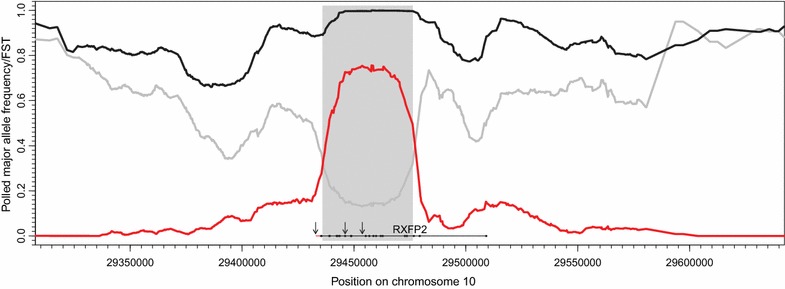
*F*_*ST*_-values and frequencies of the major allele of the sequence of the captured region. In order to define the chromosomal segment that segregates with the polled phenotype within the sequenced region obtained by sequence capture, frequencies of the polled major allele were estimated in both groups (polled vs. horned) separately and averaged over 20-kb sliding windows (*black*: polled and *gray*: horned). *F*_*ST*_-values (*red curve*) were estimated based on the allele frequencies of the same 20-kb windows. The *gray box* outlines *F*_*ST*_-values that are higher than 0.3159 (mean *F*_*ST*_-value plus one standard deviation). In addition, the position of the *RXFP2* gene is indicated as a *black line* below the curves (*thin line*: introns and *thick line*: exons). The insertion polymorphism is indicated as a *red line* on the edge of *RXFP2*. From *left* to *right*, the positions of SNPs rs421908034 (3′-UTR), rs414104606 (exon14) and OAR_29511510.1/rs413264476 (intron 11) are indicated with *arrows*

Alignment of sequence data generated from a 246-kb region that included the *RXFP2* gene and part of the flanking regions from 24 sheep samples of sample set 1 showed efficient enrichment of the target region (>100× coverage) and revealed an insertion polymorphism of about 2 kb in the 3′-UTR of *RXFP2.* Besides this manually-detected insertion polymorphism, only one SNP (A/G) at position NC_019467.2:29,432,846 (rs421908034) passed the automated filtering process and remained as a putative causal candidate based on the VarScan results. This SNP is located 214 bp away from the insertion polymorphism and Fig. 2 shows that it is positioned close to the beginning of the estimated IBD region. The insertion was absent in the 12 sheep from horned breeds, whereas among the sheep from completely polled breeds, 11 were homozygous and only a single female Pomeranian Coarsewool individual was heterozygous for the insertion. The nearby 3′-UTR SNP rs421908034 showed exactly the same genotype distribution (12 horned sheep *GG*, 11 polled sheep *AA*, and one polled Pomeranian Coarsewool sheep *AG*). Sequence data of all 24 samples were deposited in the SRA archive (http://www.ncbi.nlm.nih.gov/Traces/sra/; accession number SRP057491).

**Fig. 3:**
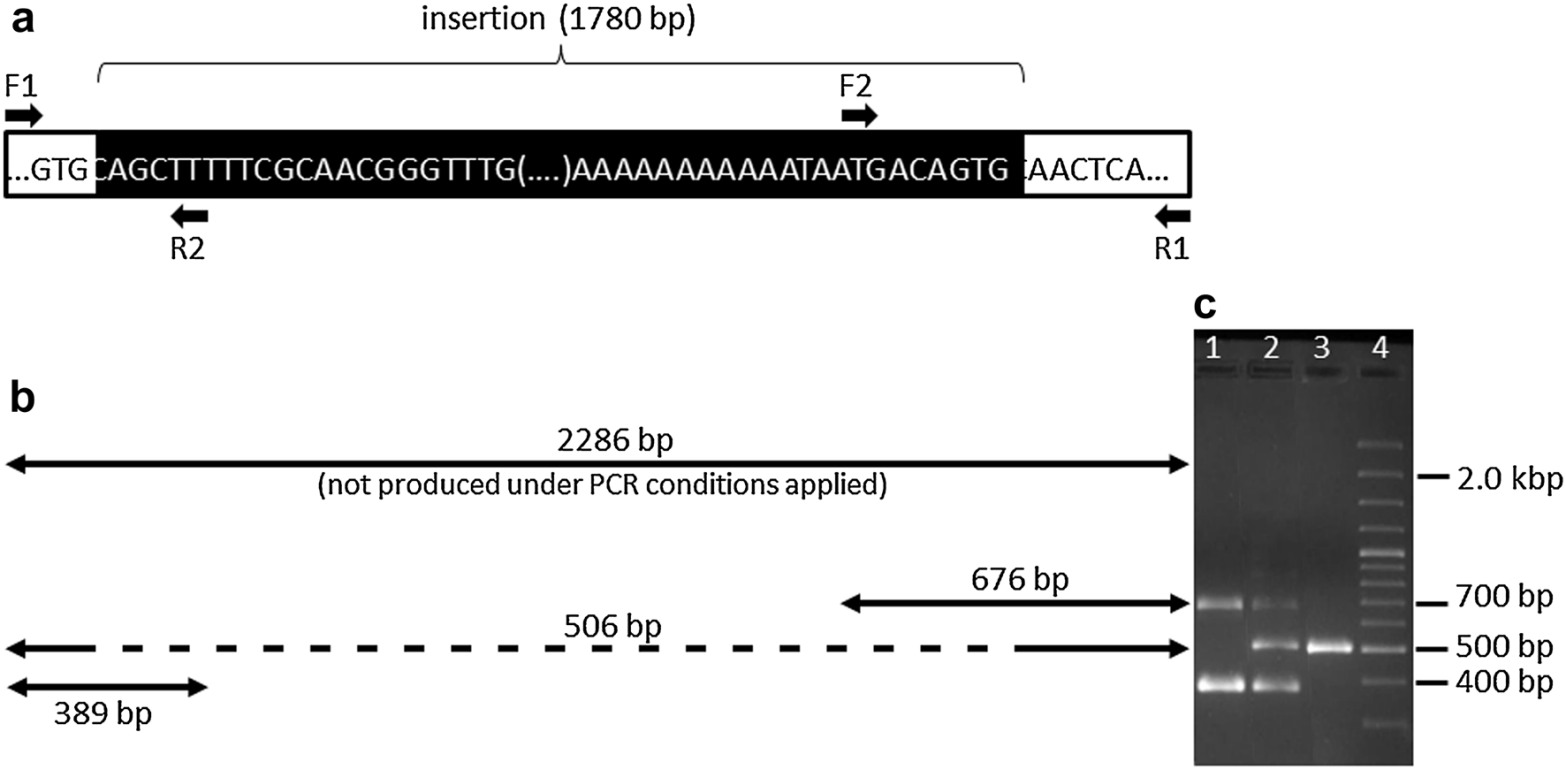
Genotyping of the ancestral (*anc*) and derived (*der*) alleles in the 3′-UTR of *RXFP2* by multiplex PCR. **a** Nucleotides at the start and end positions of the 1.78-kb insertion (derived allele) in the 3′-UTR of *RXFP2*. *Arrows* indicate the locations of the forward (F1, F2) and reverse (R1, R2) primers. **b** Regions and sizes of PCR products expected from multiplex PCR with primers F1, F2, R1 and R2. **c** Agarose gel of the PCR products obtained with primers F1, F2, R1 and R2 and DNA samples from sheep with genotypes *der*/*der* (*lane 1*), *anc*/*der* (*lane 2*) and *anc*/*anc* (*lane 3*). *Lane* 4 = DNA size marker GeneRuler™ 100 bp Plus DNA Ladder (ThermoFisher Scientific, Schwerte)

Sanger sequencing of a 2286-bp PCR fragment obtained with primer pair F1/R1 and DNA from completely horned and completely polled breeds revealed that the exact size of the insertion segregating with polledness is 1780 bp (Fig. 3a). This insertion is between positions 29, 433, 060 and 29, 434, 923 bp in the reference sequence NC_019467.2 (Oar_v4.0). This part of the ovine *RXFP2* reference sequence includes a stretch of 83 bp (NC_019467.2:29,434,159-29,434,241) that was absent from the sequence of all polled sheep analyzed in this study. In addition, all sequenced polled sheep were homozygous for 13 single nucleotide substitutions and one single nucleotide deletion compared to the reference sequence. Another homozygous nucleotide substitution that was located upstream of the 1.78-kb insertion was identified in all sequenced sheep. Both the 83- and the 1-bp deletion were also observed in polled sheep by Wiedemar and Drögemüller [24]. Sequences with and without the 1.78-kb insertion including all substitutions and deletions not present in the NC_019467.2 (Oar_v4.0) reference sequence were submitted to GenBank (http://www.ncbi.nlm.nih.gov/nucleotide; accession numbers KX084522 and KX084523).

Multiple sequence alignment of the region that surrounds the variable site in the 3′-UTR of *RXFP2* to whole-genome sequences of seven *Bovidae* showed that the 1.78-kb insertion is detected only in the sheep reference genome (which originates from a polled Texel sheep) and not in any of the reference genomes of horned *Bovidae* (see Additional file 2: Figure S1). This clearly confirmed that the 1.78-kb insertion is a derived allele. In the following sections and tables, we refer to the two alleles of the 3′-UTR of *RXFP2* as derived (*der*, with the 1.78-kb insertion) and ancestral (*anc*, without the insertion) alleles.

### Genotyping results of the 1.78-kb insertion polymorphism in the 3′-UTR *RXFP2*

The established multiplex PCR proved to be a useful tool to determine the three possible genotypes *anc*/*anc*, *anc*/*der* and *der*/*der* (Figs. 3b, c) and was used to genotype all individuals of the three sample sets. In sample set 1, 204 sheep from completely polled breeds were homozygous *der*/*der*, whereas one Pomeranian Coarsewool female and three Barbados Black Belly females were heterozygous *anc*/*der*. In contrast, all 84 sheep from completely horned breeds were homozygous *anc*/*anc*. There was no disagreement between the multiplex PCR genotypes and the 24 sequencing results (Table 1).

In sample set 2 (Table 2), the eight single crossbred sheep were the progeny of *der*/*der* sires from completely polled breeds (East Friesian or Merinolandschaf) mated with *anc*/*anc* females from the completely horned breed Grey Horned Heidschnucke. As expected, all single crossbred sheep were heterozygous *anc*/*der*. All males were horned and all females were polled. Among the 10 female progenies from multiple crosses between completely polled breeds and completely horned breeds (Table 2), all three possible genotypes were observed. Horns were observed only in sheep with the genotype *anc*/*anc*, whereas polled sheep had genotypes *der*/*der* or *anc*/*der*. However, horn rudiments were detected in a single heterozygous *anc*/*der* individual.

In contrast to our observations for completely polled and completely horned breeds and their crosses, the ancestral and derived alleles of the 3′UTR of *RXFP2* did not segregate with horn status in most of the sheep breeds of sample set 3 with sex-dependent or variable horn status (Table 3). Although all nine horned Cameroon rams were homozygous *anc*/*anc*, all 11 polled ewes of this breed were also *anc*/*anc*. Similarly, 13 polled Travnička Pramenka ewes and one polled Cres ewe were homozygous *anc*/*anc*. However, all 47 genotyped sheep of the breeds Dorper and Bovec-like were monomorphic for the derived allele, although 18 individuals (males as well as Bovec-like females) were horned and 12 additional sheep of both sexes had scurs or horn rudiments. In the Walachenschaf, Alpines Steinschaf and Bavarian Forest breeds, discrepancies regarding the segregation of ancestral and derived alleles with horn status were not as obvious as in Cameroon, Travnička Pramenka, Dorper and Bovec-like sheep. The discrepancies mostly concerned heterozygous individuals and/or the occurrence of scurs or horn rudiments in males and females (Table 3).

### Genotyping results of the SNP in the 3′-UTR *RXFP2*

Besides the 24 sheep for which sequence capture and sequencing were available, 61 sheep from sample set 3 from a breed with sex-dependent horns (Cameroon) and from two breeds with variable horn status (Walachenschaf and Dorper) were also genotyped for the SNP in the 3′-UTR of *RXFP2* (rs421908034) (see Additional file 3: Table S2). In most (59) of the 61 analyzed sheep, allele *A* segregated with the derived allele and allele *G* with the ancestral allele. Only two Cameroon sheep (one polled female and one horned male) that were heterozygous (*AG*) for this SNP but homozygous *anc/anc* were in contradiction with perfect LD between both mutations in the 3′-UTR of *RXFP2*. The presence of all three genotypes at the rs421908034 SNP in horned male sheep excluded a direct causal relationship with polledness in the overall sheep population. Therefore, genotyping of the SNP in the 3′-UTR of *RXFP2* was not pursued further in this study.

### Genotyping results for the SNP in exon 14 of *RXFP2*

The 61 sheep of the same sample set 3 were also genotyped for the SNP in exon 14 of *RXFP2* (rs414104606). As for the insertion polymorphism and the SNP in the 3′-UTR of *RXFP2*, all 24 Dorper sheep were homozygous *AA* for this SNP, although this group contained horned, polled and scurred male sheep as well as scurred female sheep (see Additional file 3: Table S2). In the Cameroon breed, the most common genotype was *AA* with some heterozygous *AG* sheep. These two genotypes were found in horned males as well as in polled females. In 13 horned Walachenschaf individuals, all three possible genotypes (*AA*, *GG* and *AG*) at SNP rs414104606 were observed. For the 24 sheep for which sequence capture and sequencing were performed, all polled animals were homozygous *AA*, 11 horned sheep were homozygous *GG*, and one horned female was heterozygous *AG*. Therefore, as for the SNP in the 3′-UTR of *RXFP2*, genotyping of the SNP in exon 14 was not pursued further.

### Genotype distribution of SNP OAR10_29511510.1 in intron 11 of *RXFP2*

**Fig. 4:**
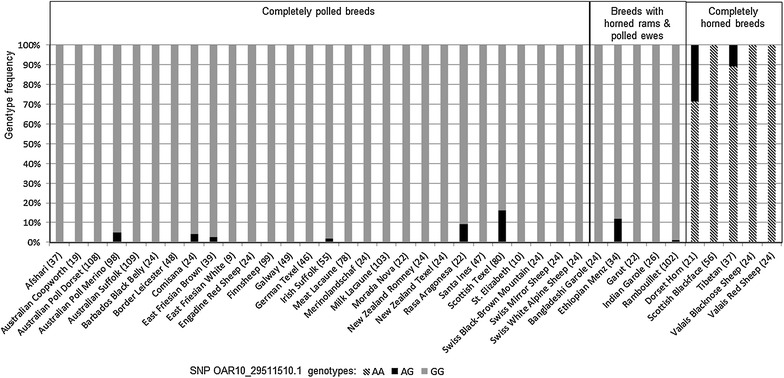
Genotype frequencies at SNP OAR10_29511510.1 for 38 international sheep breeds belonging to different horn status categories. In order to compare the genotype frequencies at SNP OAR10_29511510.1 in intron 11 of *RXFP2*, 38 breeds with reliable information about their horn status were chosen from the 74 international sheep breeds genotyped by the International Sheep Genome Consortium [22]. Assignment to one of the three horn status categories (“completely polled”, “horned rams and polled ewes” and “completely horned”), numbers of genotyped sheep (after the breed name, in brackets) and genotype frequencies (%) are provided for each breed

Among the 74 international sheep breeds that were genotyped for the SNP OAR10_29511510.1 in intron 11 of *RXFP2*, reliable information about the horn status was available for 38 breeds (28 breeds completely polled, 5 breeds with horned males and polled females, 5 breeds completely horned). Figure 4 shows the assignment to one of the three horn status groups, the number of genotyped sheep and genotype frequencies for each breed. Among the 28 completely polled breeds, all except seven breeds were monomorphic for genotype *GG*. For the seven non-monomorphic breeds, we estimated frequencies of genotype *AG* that ranged from 0.01 (Australian Suffolk) to 0.08 (Scottish Texel), but no homozygous *AA* sheep were detected. The distribution of SNP OAR10_29511510.1 genotypes was similar for breeds with horned males and polled females with three breeds having genotype *GG* and two breeds that included some individuals with genotype *AG* (*AG* frequencies of 0.01 and 0.12 were estimated in the Rambouillet and Ethiopian Menz breeds, respectively). In contrast, three of the five completely horned breeds were homozygous *AA* and no homozygous *GG* sheep were detected (Fig. 4). In the Tibetan and the Dorset Horn breeds, the frequencies of genotype *AG* were 0.11 and 0.29, respectively.

Table 4 provides the sex of the sheep that were genotyped for SNP OAR10_29511510.1 from completely polled and completely horned breeds that were not monomorphic and from all five breeds with horned males and polled females. Animals with genotype *AG* occurred in both sexes and 17 males from completely polled breeds and six females from completely horned breeds were also heterozygous *AG*. Moreover, Table 4 shows that nearly all males from breeds with horned rams and polled ewes were homozygous *GG*.

**Table 4:** Genotypes at SNP OAR10_29511510.1 for sheep from completely polled, sex-dependent horned, and completely horned sheep breeds

Horn status of breed	Breed	Sex	Genotypes (n)		
			*AA*	*AG*	*GG*
Completely polled	Australian Poll Merino	Male	0	5	93
	Australian Suffolk	Male	0	1	108
	Comisana	Female	0	5	93
	East Friesian Brown	Male	0	0	15
		Female	0	1	23
	Irish Suffolk	Male	0	1	38
		Female	0	0	16
	Rasa Aragonesa	Male	0	1	3
		Female	0	1	17
	Scottish Texel	Male	0	9	31
		Female	0	4	36
Males horned, females polled	Ethiopian Menz	Male	0	1	17
		Female	0	3	13
	Bangladeshi Garole	Male	0	0	6
		Female	0	0	18
	Garut	Male	0	0	8
		Female	0	0	14
	Indian Garole	Male	0	0	4
		Female	0	0	22
	Rambouillet	Male	0	1	75
		Female	0	0	26
Completely horned	Dorset horn	Male	3	1	0
		Female	12	5	0
	Tibetan	Male	19	3	0
		Female	14	1	0

*n* number

## Discussion

### Segregation of the 1.78-kb insertion polymorphism in the 3′-UTR of *RXFP2* with horn status

Targeted sequencing of the *RXFP2* gene and its flanking regions in 24 sheep from 12 different either completely polled or completely horned breeds allowed us to identify the same insertion polymorphism within the 3′-UTR of *RXFP2* that was recently shown to segregate with horn status in Swiss breeds by Wiedemar and Drögemüller [24]. Sequence comparisons with other horned *Bovidae* confirmed this insertion as the derived allele. In both studies, completely horned breeds were shown to be fixed for the ancestral allele without the insertion in the 3′-UTR of *RXFP2*. In contrast to the Swiss study in which all sheep from completely polled breeds were monomorphic for the insertion, we detected four ewes from completely polled breeds (Barbados Black Belly and Pomeranian Coarsewool) that carried the insertion on only one chromosome. However, under the hypothesis that horns are dominant in the male and recessive in the female [7], heterozygous ewes are expected to be polled and hence in agreement with the breed’s standard horn status. The relative high proportion of heterozygous sheep (3 out of 10) among the Barbados Black Belly sheep may not be representative since all investigated samples of this breed originated from a single small breeding flock and thus the ewes were related to each other.

A sex-limited effect of the derived allele became even clearer after genotyping sheep that originated from single or multiple crosses between completely polled breeds and completely horned breeds: male sheep with the genotype *anc*/*der* were horned, whereas female sheep with this genotype were polled. This is in agreement with previous studies on crossed sheep [6–8]. However, the fact that we identified a single female sheep with horn rudiments, which originated from multiple crosses between polled and horned breeds, and a polled Cameroon ram (atypical for this breed) that both had the genotype *anc*/*der* disputed complete association (Tables 2, 3).

Similarly, two sheep breeds that originated from Southern Germany (Alpines Steinschaf and Bavarian Forest) further challenged the segregation of the insertion in the 3′-UTR of *RXFP2* with the polled phenotype (Table 3). In summary, for these breeds both the effect of the *anc*/*der* genotype on the one hand and the scurs/horn rudiments phenotype on the other hand showed no regular pattern. In addition, we observed one male Alpines Steinschaf with scurs that was homozygous for the *der*/*der* genotype.

Among our samples, those that originated from even more southern European regions (Bovec-like sheep, Cres sheep, Travnička Pramenka and Walachenschaf) as well as from Africa (Dorper and Cameroon sheep) provided additional evidence that challenged a causal relationship or segregation of the insertion polymorphism in the 3′-UTR of *RXFP2* with horn status. In these breeds, we observed almost all possible combinations of genotypes, sex and horn status phenotypes (Table 3). Only polled males with the *anc*/*anc* genotype were not observed, which could be due to the lower proportion of sampled polled males (10 %) in the three relevant Southern European sheep breeds.

Based on our results, the insertion polymorphism in the 3′-UTR of *RXFP2* appears to segregate with horn status only in certain sheep breeds, such as the completely polled and completely horned breeds, and (up to a certain degree) in some breeds with variable horn status. In other breeds with variable horn status in both sexes or with a sex-dependent horn status, this segregation is not observed. Furthermore, our results suggest that the perfect LD between the insertion polymorphism and the horn phenotype is gradually eroded in the Southeast European and African breeds as the spatial distance to the North and Central European breeds with fixed horn status increases. However, the absence of *anc*/*anc* polled males should not be considered as trivial because without this combination, we cannot definitely exclude the 1.78-kb insertion as one of maybe several causal contributors to the complex polled phenotype in sheep.

### Segregation of the SNPs in the 3′-UTR and exon 14 of *RXFP2* with horn status

In addition to the genotypes that were obtained by sequence capture and sequencing of 24 sheep, both candidate SNPs (rs421908034 and rs414104606) were also genotyped in 61 sheep from breeds with variable and sex-dependent horn statuses. As shown in Table S2 (see Additional file 3: Table S2), LD between these two SNPs and the 1.78-kb insertion is nearly perfect. Allele *G* of SNP rs421908034 in the 3′-UTR of *RXFP2* forms a haplotype with the ancestral allele (without the insertion), whereas allele *A* is linked with the derived allele (with the insertion). Results for only two Cameroon sheep disagree with a perfect LD between these two closely located (214 bp) mutations. The synonymous SNP (rs414104606) in exon 14 of *RXFP2* is located further away (11.1 kb) from the insertion in the 3′UTR of this gene. Consequently, genotyping results suggest an additional deviation from perfect LD between both candidate mutations. Genotyping results showed that Dorper sheep were monomorphic for genotype *AA*, although horned, scurred and polled phenotypes were involved. Moreover, segregation of SNP rs414104606 with horn status was not observed in the two other genotyped breeds with variable (Walachenschaf) and sex-dependent (Cameroon sheep) horn statuses. In addition, the alleles of SNP rs414104606 and the insertion polymorphism in the 3′-UTR of *RXFP2* segregated predominantly in opposite phases in these two breeds (see Additional file 3: Table S2). In accordance with our results, Wang et al. [23] also observed that sheep from a completely polled breed (Suffolk) carried genotype *AA* at SNP rs414104606. They also identified a polled female Han sheep with genotype *GG*, which adds another sheep breed with variable horn status to the group of breeds in which this SNP does not segregate with horn status.

### Segregation of SNP OAR10_29511510.1 with horn status

By re-analyzing SNP genotyping data provided by the ISGC, we made the same observations for SNP OAR10_29511510.1 in the intron of *RXFP2*. On the one hand, this SNP segregated perfectly with horn status in 21 completely polled and in three completely horned breeds (polled: *GG*, horned: *AA*). According to our observation that horn status is sex-dependent in polled and completely horned sheep breeds that are heterozygous for the insertion polymorphism in the 3′-UTR of *RXFP2*, sheep that are heterozygous for SNP OAR10_29511510.1 would fit their breed’s standard horn status only if all heterozygous animals were either female (polled breeds) or male (horned breeds). However, among the completely polled breeds, five breeds included males with genotype *AG* and two completely horned breeds included females with genotype *AG* (Table 4). Even more significant was the identification of 110 male sheep with genotype *GG* at SNP OAR10_29511510.1 from five breeds with horned males and polled females (Table 4). If the genotype *GG* segregates with polledness, those males would be expected to be polled. This clearly contrasts with their breed standard that defines males as consistently horned. Admittedly, the individual horn status of these sheep was not known. Thus, it is possible that some of the sheep included in the data that we used did not comply with the standard horn status of their respective breeds (e.g. it is known that in polled breeds, sheep with knobs, scurs or even normal horns occasionally occur). However, this would not explain the large number of cases for which the genotype at OAR10_29511510.1 and the breed-specific horn status did not segregate, e.g. as observed for 75 (98.7 %) males from the sex-dependent horned Rambouillet breed (Table 4).

In order to analyze LD between SNP OAR10_29511510.1 and the insertion polymorphism in the 3′-UTR of *RXFP2*, we genotyped 75 sheep from six completely polled and six completely horned breeds (n = 32) and from four breeds with variable horn status (n = 43) using available capture sequencing data from this study or Sanger sequencing, and the multiplex PCR developed in this study (details on the breeds included and genotyping data not shown). Although the resulting estimated LD (R package *genetics*) was relatively low over all 75 sheep (r^2^ = 0.369), it was 3.27 times higher in the 32 sheep from completely polled and completely horned breeds (r^2^ = 0.635) than in the 43 sheep from breeds with variable horn status (r^2^ = 0.194), which suggests a strong association of this SNP in mapping designs that include completely polled and completely horned breeds and a weak association in designs that include sheep from breeds with variable horn status.

### Possible reasons for breed differences in the segregation of variants within *RXFP2* with horn status

Wiedemar and Drögemüller [24] postulated that the insertion in the 3′-UTR of the *RXFP2* gene had an effect on the processing and translation of this gene and therefore inhibited normal horn growth. Interestingly, the insertion was predicted to be a complete protein coding gene (*LOC101110773*) by automated computational analysis using Gnomon (http://www.ncbi.nlm.nih.gov/nuccore/NC_019467.2?report=genbank&from=29433047&to=29434884), which agrees with the results of Pickering et al. [20] that indicated a retrotransposition of a complete functional mRNA during the insertion event. The *LOC101110773* gene is predicted to encode an elongation factor 1-alpha 1-like protein, which may modify the tissue-specific transcription of *RXFP2* or other loci. Quite different from the prediction based on the reference sheep genome Oar_v3.1, only a short 3′UTR (without an insertion) is predicted for *RXFP2* based on Oar_v4.0, and thus, the complete 1.78-kb insertion is assigned to the next neighboring gene, i.e. *LOC101110773*. Taking this fact into account, there are different important questions that need to be answered: (1) are the 3′UTR of the *RXFP2* mRNA in polled and horned sheep with *der/der* and *anc/anc* genotypes different?, (2) assuming that there are no differences in the mRNA, can differential expression of the *LOC101110773* gene be involved?, and (3) could SNP rs421908034, which is positioned 214 bp away from *LOC101110773,* influence its expression and thus indirectly modify the transcription of *RXFP2*? The design used in our study is not suitable for answering these and other similar questions but provides information for an improved future mapping design, differential expression and gene editing studies. However, according to our observations, to explain an effect of the 1.78-kb insertion, it would be necessary to involve complementary causality of one or more other, currently unknown, variants in the sheep genome. This complementary causality of more alleles or genes has been speculated since the 1940s (see e.g. [5]). Nonetheless, all recent designs for genome-wide association analyses pointed towards a very strong effect of a single locus that is near or within *RXFP2* [14]. Our results (Fig. 2) also strongly suggest a signature of positive selection in the completely polled breeds of Central and Western European origin. This selection signature is most prominent in an interval of ~40 kb (between 29,436,000 and 29,476,000 bp). Therefore, it is reasonable to expect some causal relationships between polledness and the observed genetic variation within this region. However, although considerable efforts were made in this study to ensure comprehensive information and results, we still cannot exclude the presence of some other variants or combinations of variants within *RXFP2* that might trigger alternative splicing or similar mechanisms thus contributing to the polled phenotype. Therefore, there is still the possibility that the 1.78-kb insertion polymorphism has no causal effect on horn status and is only in strong LD with one or more unknown causal variants. In this context, the differences in segregation of the 1.78-kb insertion with horn status in various breeds could be due to allelic heterogeneity and to the presence of some currently unknown, breed-specific causal alleles with different levels of LD with the 1.78-kb insertion polymorphism and other polymorphisms near or within *RXFP2*. It is very likely that at least one of the complementary causal variants is in interaction with or located on the sex chromosomes. In this context, it should be mentioned that *RXFP2* codes for the receptor of the insulin-like peptide 3 (INSL3) that is a major secretory product of the testicular Leydig cells of male mammals [27] and seems, among others, to play an important role in the fetal abdominal *descensus testis* and in maintaining spermatogenesis [28].

## Conclusions

Based on our findings, we conclude that the insertion polymorphism in the 3′-UTR of *RXFP2* and the SNPs in the 3′-UTR, exon 14 and intron 11 of this gene (some of these previously shown to be associated with horn status in several sheep breeds) are, at least in breeds with a variable and/or sex-dependent horn status, not in population-wide LD with the horn status and thus cannot be used as universal markers to define the genetic horn status in the global sheep population. Moreover, the 1.78-kb insertion can be excluded as the only cause of polledness in sheep. Our results suggest that future studies aimed at solving this question should focus on breeds with variable and sex-dependent horn status. To exploit historical recombinations, emphasis should be placed on collecting samples and precise phenotypes from breeds with larger spatial and genetic distance to breeds that originate from Northern and Central Europe. Moreover, the procedures should be able to fulfill the requirements for mapping under the hypothesis of a heterogeneous and/or polygenic mode of inheritance. Similarly, in order to increase the probability of success, the design of future differential expression, alternative splicing and gene editing studies should consider various combinations of neighboring variants such as the SNPs and the insertion in the 3′-UTR of *RXFP2*.

## Additional files

**Additional file 1: Table S1.** Primers used for Sanger sequencing and genotyping the insertion in the 3’-UTR of *RXFP2.*

**Additional file 2: Figure S1.** Multiple sequence alignment of the region surrounding the insertion in the 3’-UTR of *RXFP2*. The 506-bp sequence amplified in sheep from horned breeds with primers F1/R1 was searched in the whole genome sequence of seven *Bovidae* using NCBI’s blast server (https://blast.ncbi.nlm.nih.gov/Blast.cgi. Accessed 11 April 2016) with default settings (Megablast). The results are schematically shown by plotting the obtained alignments as grey rectangles and by highlighting SNPs and small indels with white vertical bars. The 1.78-kb insertion is only found in the sheep reference genome (that originates from a polled Texel sheep) but not in any of the reference genomes of horned *Bovidae*. The sequence is highly conserved in bovids (93 to 99 % sequence identity), in contrast to other mammals that only show a maximum of 78 % sequence identity (not shown).

**Additional file 3: Table S2.** Genotypes at the insertion polymorphism in the 3’-UTR of *RXFP2*, at SNP rs421908034 in the 3’-UTR and SNP rs414104606 in exon 14 of *RXFP2* for 61 sheep from three breeds with variable and sex-dependent horn status.
