# Supplementary material for: The 1.78-kb insertion in the 3′-untranslated region of RXFP2 does not segregate with horn status in sheep breeds with variable horn status
Source: Genet Sel Evol. 2016 Oct 19;48:78. doi: 10.1186/s12711-016-0256-3 (PMC5072343; doi:10.1186/s12711-016-0256-3)
Supplement: Supplementary file 1 — Additional file 1: Table S1. Primers used for Sanger sequencing and genotyping the insertion in the 3’-UTR of RXFP2. [file 12711_2016_256_MOESM1_ESM.pdf]

Additional Table S1

Primer name	Primer sequence (5’-3’)	Primer position OAR10_v4.0
F1	GAGGCCTTGAGGTGGAAGTC	29,432,881-29,432,900
F2	TGCCCAGAAAGCTCAGAAGG	29,434,574-29,434,593
R1	TGGAGCACTGGGCTTCAAAT	29,435,231-29,435,250
R2	GCTGCCTCCTTCTCGAACTT	29,433,250-29,433,269
F3	AGCCAGAAGAGATACGAGGAAAT	29,433,616-29,433,638
F4	GGGATGGAAAGTCACCCGTA	29,433,765-29,433,784
