# Supplementary figures and images for: The 1.78-kb insertion in the 3′-untranslated region of RXFP2 does not segregate with horn status in sheep breeds with variable horn status
Source: Genet Sel Evol. 2016 Oct 19;48:78. doi: 10.1186/s12711-016-0256-3 (PMC5072343; doi:10.1186/s12711-016-0256-3)

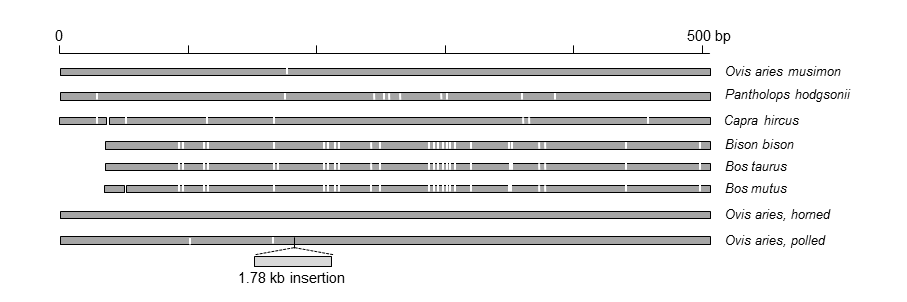

Supplement: Supplementary file 2 — Additional file 2: Figure S1. Multiple sequence alignment of the region surrounding the insertion in the 3’-UTR of RXFP2. The 506-bp sequence amplified in sheep from horned breeds with primers F1/R1 was searched in the whole genome sequence of seven Bovidae using NCBI’s blast server (https://blast.ncbi.nlm.nih.gov/Blast.cgi. Accessed 11 April 2016) with default settings (Megablast). The results are schematically shown by plotting the obtained alignments as grey rectangles and by highlighting SNPs and small indels with white vertical bars. The 1.78-kb insertion is only found in the sheep reference genome (that originates from a polled Texel sheep) but not in any of the reference genomes of horned Bovidae. The sequence is highly conserved in bovids (93 to 99 % sequence identity), in contrast to other mammals that only show a maximum of 78 % sequence identity (not shown). [file 12711_2016_256_MOESM2_ESM.png]
