# Supplementary material for: The 1.78-kb insertion in the 3′-untranslated region of RXFP2 does not segregate with horn status in sheep breeds with variable horn status
Source: Genet Sel Evol. 2016 Oct 19;48:78. doi: 10.1186/s12711-016-0256-3 (PMC5072343; doi:10.1186/s12711-016-0256-3)
Supplement: Supplementary file 3 — Additional file 3: Table S2. Genotypes at the insertion polymorphism in the 3’-UTR of RXFP2, at SNP rs421908034 in the 3’-UTR and SNP rs414104606 in exon 14 of RXFP2 for 61 sheep from three breeds with variable and sex-dependent horn status. [file 12711_2016_256_MOESM3_ESM.pdf]

Additional Table S2

Sheep No.	Breed	Horn status of anima	Sex	Genotypes (n) of *RXFP2* 3'-UTR insertion poly-morphism	3'-UTR SNP[1]	Exon 14 SNP[2]
1	Dorper	polled	male	*der*	*AA*	*AA*
2	Dorper	polled	male	*der*	*AA*	*AA*
3	Dorper	polled	male	*der*	*AA*	*AA*
4	Dorper	polled	male	*der*	*AA*	*AA*
5	Dorper	polled	male	*der*	*AA*	*AA*
6	Dorper	polled	male	*der*	*AA*	*AA*
7	Dorper	horned	male	*der*	*AA*	*AA*
8	Dorper	horned	male	*der*	*AA*	*AA*
9	Dorper	horned	male	*der*	*AA*	*AA*
10	Dorper	horned	male	*der*	*AA*	*AA*
11	Dorper	horned	male	*der*	*AA*	*AA*
12	Dorper	horned	male	*der*	*AA*	*AA*
13	Dorper	horned	male	*der*	*AA*	*AA*
14	Dorper	horned	male	*der*	*AA*	*AA*
15	Dorper	scurred	male	*der*	*AA*	*AA*
16	Dorper	scurred	male	*der*	*AA*	*AA*
17	Dorper	scurred	male	*der*	*AA*	*AA*
18	Dorper	scurred	female	*der*	*AA*	*AA*
19	Dorper	scurred	female	*der*	*AA*	*AA*
20	Dorper	scurred	female	*der*	*AA*	*AA*
21	Dorper	scurred	female	*der*	*AA*	*AA*
22	Dorper	scurred	female	*der*	*AA*	*AA*
23	Dorper	scurred	female	*der*	*AA*	*AA*
24	Dorper	scurred	female	*der*	*AA*	*AA*
1	Walachenschaf	horned	male	*anc*	*GG*	*GG*
2	Walachenschaf	horned	male	*anc*	*GG*	*AG*
3	Walachenschaf	horned	male	*anc/der*	*AG*	*AA*
4	Walachenschaf	horned	male	*anc*	*GG*	*GG*
5	Walachenschaf	horned	male	*anc*	*GG*	*GG*
6	Walachenschaf	horned	female	*anc*	*GG*	*AG*
7	Walachenschaf	horned	female	*anc*	*GG*	*AG*
8	Walachenschaf	horned[3]	female	*anc/der*	*AG*	*AG*
9	Walachenschaf	horned	female	*anc*	*GG*	*AG*
10	Walachenschaf	horned[3]	female	*anc*	*GG*	*GG*
11	Walachenschaf	horned	female	*anc*	*GG*	*GG*
12	Walachenschaf	horned[3]	female	*anc*	*GG*	*GG*
13	Walachenschaf	horned	female	*anc*	*GG*	*GG*
1	Cameroon	polled	female	*anc*	*GG*	*AA*
2	Cameroon	polled	female	*anc*	*GG*	*AA*
3	Cameroon	polled	female	*anc*	*GG*	*AA*
4	Cameroon	horned	male	*anc*	*GG*	*AA*
5	Cameroon	horned	male	*anc*	*GG*	*AA*
6	Cameroon	horned	male	*anc*	*GG*	*AA*
7	Cameroon	horned	male	*anc*	*GG*	*AA*

Sheep No.	Breed	Horn status of anima	Sex	Genotypes (n) of *RXFP2*<br>3'-UTR insertion poly-morphism	3'-UTR SNP[1]	Exon 14 SNP[2]
8	Cameroon	horned	male	*anc*	*GG*	*AG*
9	Cameroon	polled	female	*anc*	*GG*	*AG*
10	Cameroon	polled	female	*anc*	*GG*	*AA*
11	Cameroon	polled	female	*anc/der*	*AG*	*AA*
12	Cameroon	polled	female	*anc*	*GG*	*AA*
13	Cameroon	polled	female	*anc*	*GG*	*AA*
14	Cameroon	polled	female	*anc*	*GG*	*AA*
15	Cameroon	polled	female	*anc*	*AG*	*AG*
16	Cameroon	polled	female	*anc/der*	*AG*	*AG*
17	Cameroon	polled	female	*anc*	*GG*	*AA*
18	Cameroon	horned	male	*anc*	*GG*	*AA*
19	Cameroon	horned	male	*anc*	*GG*	*AA*
20	Cameroon	horned	male	*anc*	*GG*	*AA*
21	Cameroon	horned	male	*anc*	*AG*	*AG*
22	Cameroon	polled	male	*anc/der*	*AG*	*AA*
23	Cameroon	horned	male	*anc*	*GG*	*AA*
24	Cameroon	horned	male	*anc*	*GG*	*AA*

anc = ancestral allele; *der* = derived allele (with 1780 bp-insertion); [1)](rs421908034); [2](rs414104606); [3)]horn rudiments.
